# Characterization of Bone Marrow-Derived Dendritic Cells Developed in Serum-Free Media and their Ability to Prevent Type 1 Diabetes in Nonobese Diabetic Mice

**DOI:** 10.4172/2155-9864.1000206

**Published:** 2014-03-22

**Authors:** Ben M Looney, Anna V Chernatynskaya, Michael J Clare-Salzler, Chang-Qing Xia

**Affiliations:** Department of Pathology, Immunology and Laboratory Medicine, University of Florida, USA

**Keywords:** Bone marrow-derived dendritic cells, Type 1 diabetes, Serum-free media, Fetal bovine serum, NOD mouse

## Abstract

Dendritic cells (DC) have been investigated as a cell-based therapy for Type 1 Diabetes (T1D). BM-DC expanded *ex vivo* with GM-CSF and IL-4 is typically cultured with fetal bovine serum (FBS). The effect of FBS on NOD BM-DC has not been extensively studied. In the present study we compare BM-DC generated in serum-free culture media (X-VIVO20; FBS−) with BM-DC generated in media containing 10% FBS (RPMI1640/10%FBS; FBS+). We show that FBS− BM-DC display a phenotype and cytokine-producing profile distinct from FBS+ BMDC. Additionally, compared to FBS+ BM-DC, we show evidence of an altered T_h_ cell response induced by FBS− BM-DC. Finally, we demonstrate that only FBS− BM-DC prevent the onset of T1D and induce increased levels of CD4^+^Foxp3^+^ regulatory T cells as well as a long-lasting β cell-specific T cell response. This study indicates that serum-free media generates a more tolerogenic BM-DC capable of preventing T1D in the NOD mice.

## Introduction

In Type 1 Diabetes (T1D), the use of recombinant insulin to maintain normoglycemia does not address the underlying cause of the disease, T cell mediated β cell destruction. T1D dendritic cell (DC) therapy addresses this issue by promoting β cell specific self-tolerance via modification of the T cell response. The use of DC to circumvent T1D progression and promote β cell self-tolerance was first investigated approximately 2 decades ago. DC from the pancreatic draining lymph nodes of non-obese diabetic (NOD) mice prevented the onset of T1D when adoptively transferred to NOD mice at an early stage of the disease [1]. Since that time numerous studies have investigated the effectiveness of bone marrow derived dendritic cells (BM-DC) in preventing T1D in NOD mice [2–6]. These studies have demonstrated that DC therapy efficacy is directly related to the phenotype and cytokine production of DC as well as the related modification of T cell responses.

Feili-Hariri et al. utilized GM-CSF and IL-4 to expand DC from bone marrow precursor cells *ex vivo* and demonstrated their ability to effectively prevent T1D onset [2]. These and other investigations have revealed that GM-CSF/IL-4 BM-DC treatment initiates a protective T_h_2 response [2–4,7]. Furthermore, evidence suggests that the expression of co-stimulatory molecules and production of TGFβ are related to the ability of BM-DC to elicit immunoregulatory responses and inhibit autoimmune disease [5,8,9]. Though this evidence supports the use of BM-DC to prevent T1D, more work is needed to validate the strategy prior to translation into the clinic. In particular, evidence suggests that various BM-DC culture conditions may have differing effects on the phenotype of the DC and, as a consequence, their ability to protect from T1D [4,10–14].

Much of the research focusing on T1D DC therapy has utilized GM-CSF/IL-4 derived BM-DC generated in the presence of fetal bovine serum (FBS) [2–6]. The inclusion of FBS results in BM-DC with phenotypic and functional differences including co-stimulatory marker expression, cytokine production and T cell stimulatory capacity [4,10–14]. Two approaches have been utilized to circumvent issues associated with the use of FBS; culture of BM-DC using serum free [10–12] or autologous serum conditioned media [4,13,14]. We chose to focus our efforts on the utilization of chemically defined serum free media to culture BM-DC as this method would provide the least resistance to translation of DC therapy into the clinic.

Though there is evidence supporting the use of serum-free media, a direct comparison of serum free and FBS cultured NOD BM-derived DC has not been undertaken. In the present study we defined the phenotype and cytokine production of each of these BM-DC populations as well as their ability to stimulate T cells and induce specific T_h_ cell cytokine profiles. Lastly, we determined the ability of each of these BM-DC populations to prevent the onset of T1D development when treatment began at a clinically relevant disease stage. We found that the phenotypic profile of BM-DC generated in serum free media are more tolerogenic and provide increased protection from T1D onset.

## Materials and Methods

### Generation of bone marrow derived dendritic cells

BM-DC was generated from 4–8 week old female NOD mice following an established protocol for the generation of immature myeloid DC. The femur and tibia were excised and flushed with phosphate buffered saline (PBS). Red blood cells were removed by treatment with ammonium chloride potassium (ACK) lysis buffer for 2 min at room temperature followed by washing with PBS. The resulting cells were cultured at a density of 1×10^6^ cells/ml in either X-VIVO20 media (FBS; Lonza, Basel, Switzerland) or RPMI1640 media (Mediatech Inc., Manassas, VA) supplemented with 10% FBS (FBS+; Hyclone Laboratories, Rockford, IL). Cultures were further supplemented with 1% penicillin/streptomycin and treated with 1000U/ml rmGM-CSF and 500U/ml rmIL-4 (R&D Systems, Minneapolis, MN). Half of the media was removed and replaced with fresh media containing GM-CSF and IL-4 every 2–3 days. By day 5 of the culture period, greater than 80% of the resulting cells expressed the myeloid cell lineage marker CD11b. BM-DC cultured in FBS− media resulted in 12–15% dead cells compared to 7–10% in FBS+ media as measured by AnnexinV/PI staining. For the investigation of BM-DC cytokine production, as well as all DC-T cell assays and DC treatment protocols, dead cells were removed by Ficoll density-gradient separation and resulting cells were enriched for CD11c+ cells using CD11c Microbeads (Miltenyi Biotec, Bergisch Gladbach, Germany). Dead cells comprised less than 5% of the resulting CD11c+ BM-DC from both FBS+ and FBS− culture conditions as evaluated by Trypan blue dye exclusion analysis.

### Flow cytometry

On day 5–6 of BM-DC culture, cells were gently removed and stained with the following antibodies (or appropriate isotype controls): CD16/CD32 Fc block (BD Biosciences, San Jose, CA), CD11c-APC (BD Biosciences), CD11c-PE-Cy7 (BD Biosciences), CD11b-PE (BD Biosciences), I-Ad-FITC (BD Biosciences), CD86-APC (BioLegend, San Diego, CA) or CD80-PE (BD Biosciences). Mean Fluorescence Intensity (MFI) of the above markers was determined on CD11c^+^ gated cells. On day 7, Leukocyte Activation Cocktail (PMA/Ionomycin/Golgi-plug; BD Biosciences) was added to culture media for the final 4 hours of culture. Cells were gently removed and stained with CD16/CD32 Fc block followed by CD11c-PE-Cy7. Cells were permeabilized and fixed (eBioscience, San Diego, CA) and then stained with anti-IL-12-PE (BD Biosciences) or anti-IL-6-PE (BD Biosciences).

### DC cytokine expression

5×10^5^ CD11c^+^ DC from both culture conditions were incubated in 200 µl of each culture media for 24 hours. Media was collected and analyzed for the presence of the indicated cytokines using a Milliplex MAP Cytokine Immunoassay (Millipore, Billerica, MA).

### *In vitro* antigen specific T cell proliferation and cytokine production

CD4^+^ T cells were isolated from spleens of BDC2.5 NOD females using CD4 Microbeads (Miltenyi Biotec). 1×105 BDC2.5 CD4+ T cells were co-cultured with 1×10^4^ CD11c+ DC in the presence of the BDC 2.5 mimepitope 1040-55 (10µg/ml) using both culture media in triplicate. On day 3 of co-culture, 50µl media was removed and analyzed for the presence of the indicated cytokines using a Milliplex MAP Cytokine Immunoassay (Millipore). 50 µl fresh media containing 1 µCi^[3]^H-Thymidine was added to the culture and incubated for 18 hours prior to cell harvest and analysis of Thymidine uptake.

### *In vivo* DC induced T cell proliferation

CD11c^+^ DC were pulsed with GAD217-236 peptide (10 µg/ml) for 1 hour at 37°C and washed twice with PBS. Spleen cells were isolated from wild type NOD females and stained with CFSE. Simultaneously, 1×10^6^ DC were injected subcutaneously in the hind footpad and 20×10^6^ CFSE labeled spleen cells were injected intravenously into NOD females. Day 5 post-injection, local (popliteal) and distal (axial) lymph node cells were isolated and stained with CD4-PerCP (BD Biosciences) and CFSE dilution was assessed via flow cytometry.

### BM-DC treatment and T1D diagnosis

BM-DCs were generated as described above and CD11c^+^ DC were pulsed with GAD217-236 peptide as previously mentioned. 1×10^5^ DC (or PBS) was injected intravenously into 8–9 week old NOD females once per week for 5 weeks. Mice were sacrificed 2 weeks following termination of treatment (n=3) or were monitored for T1D onset for 20 weeks (n=12). Bi-weekly urinalysis (Clinistix; Bayer, Tarrytown, NY) was used to detect onset of T1D. Non fasting plasma glucose levels greater than 250 mg/dl for two consecutive days positively validated T1D onset.

### Regulatory T cell analysis

Pancreatic draining lymph nodes and inguinal lymph nodes were collected from BM-DC treated mice 2 weeks following termination of treatment. Lymph node cells were stained with CD4-PerCP (BD Biosciences), permeabilized and fixed (eBioscience, San Diego, CA) and then stained with FoxP3-PE (BioLegend). CD4^+^Foxp3^+^ T_regs_ were examined by flow cytometry (BD Bioscience LSRFortessa).

### Quantification of cytokine producing T cells by ELISpot

Culture plates were pre-coated overnight with indicated capture antibodies (BD Biosciences). Spleens were collected from BM-DC treated mice 2 weeks following termination of treatment. 3×10^5^ total spleen cells were cultured in HL-1 media (Lonza) and stimulated with GAD217-236 peptide (10 µg/ml) for 4 days. Cells were removed and plates were probed with appropriate detection antibodies (BD Biosciences). Total spot forming units were enumerated with a BioReader automated spot counter (BioSys GmbH, Karben, Germany).

### Splenocyte proliferation and cytokine production

Twenty weeks following termination of BM-DC treatment, spleens were collected from remaining non diabetic mice. 1×10^6^ total spleen cells were cultured in HL-1 media in the presence of GAD217-236 peptide (10 µg/ml). On day 4, 50 µl of media was removed and analyzed for the presence of the indicated cytokines using a Milliplex MAP Cytokine Immunoassay (Millipore). 50 µl fresh media containing 1µCi ^[3]^H-Thymidine was added to the culture and incubated for 16 hours prior to cell harvest and analysis of Thymidine uptake.

### Statistical analysis

P values were calculated with a Student’s *t* test (*) or Gehan-Breslow-Wilcoxon Test (**). P values less than 0.05 were considered significant.

## Results

### Determination of BM-DC phenotype and cytokine production

**Figure 1:**
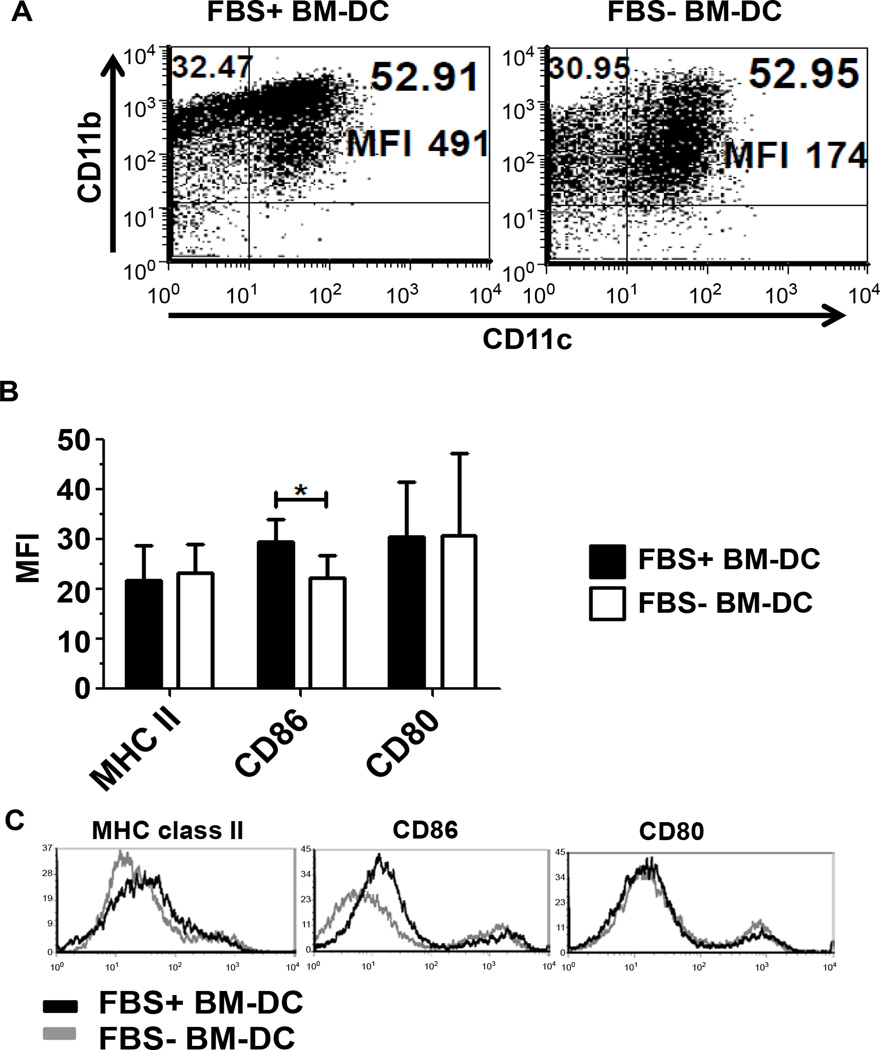
Flow cytometric profile of GM-CSF and IL-4 conditioned BM-DC. BM-DC cultured in FBS− and FBS+ conditioned media were collected on Day 5 of culture and their phenotype assessed. **A.** The expression of CD11c and CD11b on BM-DC was assessed. **B.** The Mean Fluorescence Intensity (MFI) of co-stimulatory molecules CD86 and CD80, and MHC class II was assessed in three independent experiments (n=3). **C.** Representative histograms of the indicated markers gated on CD11c^+^ cells.

The expression of the classical myeloid DC markers CD11c and CD11b were used to determine the phenotype of BM-DC from FBS− (X-VIVO20) and FBS+ (RPMI1640/10% FBS) culture conditions. In both conditions, approximately 50% of the total cells expressed both CD11c and CD11b (Figure 1A). However, on a per cell basis, CD11b expression was elevated in FBS+ BM-DC compared to FBS− BM-DC (Figure 1A). To assess the tolerance inducing capacity of the BM-DC we further determined the expression of MHC and co-stimulatory markers. On day 5 of the culture, CD11c-gated cells were analyzed for expression of MHC class II, CD86 and CD80. BM-DC from both culture conditions expressed similar levels of MHC class II suggesting a comparable ability to present antigen to CD4 T cells (Figure 1B and C). Additional characterization indicated that FBS+ BM-DC express increased CD86 relative to FBS− BM-DC, however, we observed no difference between DCs in the expression of CD80 (Figure 1B and C).

**Figure 2:**
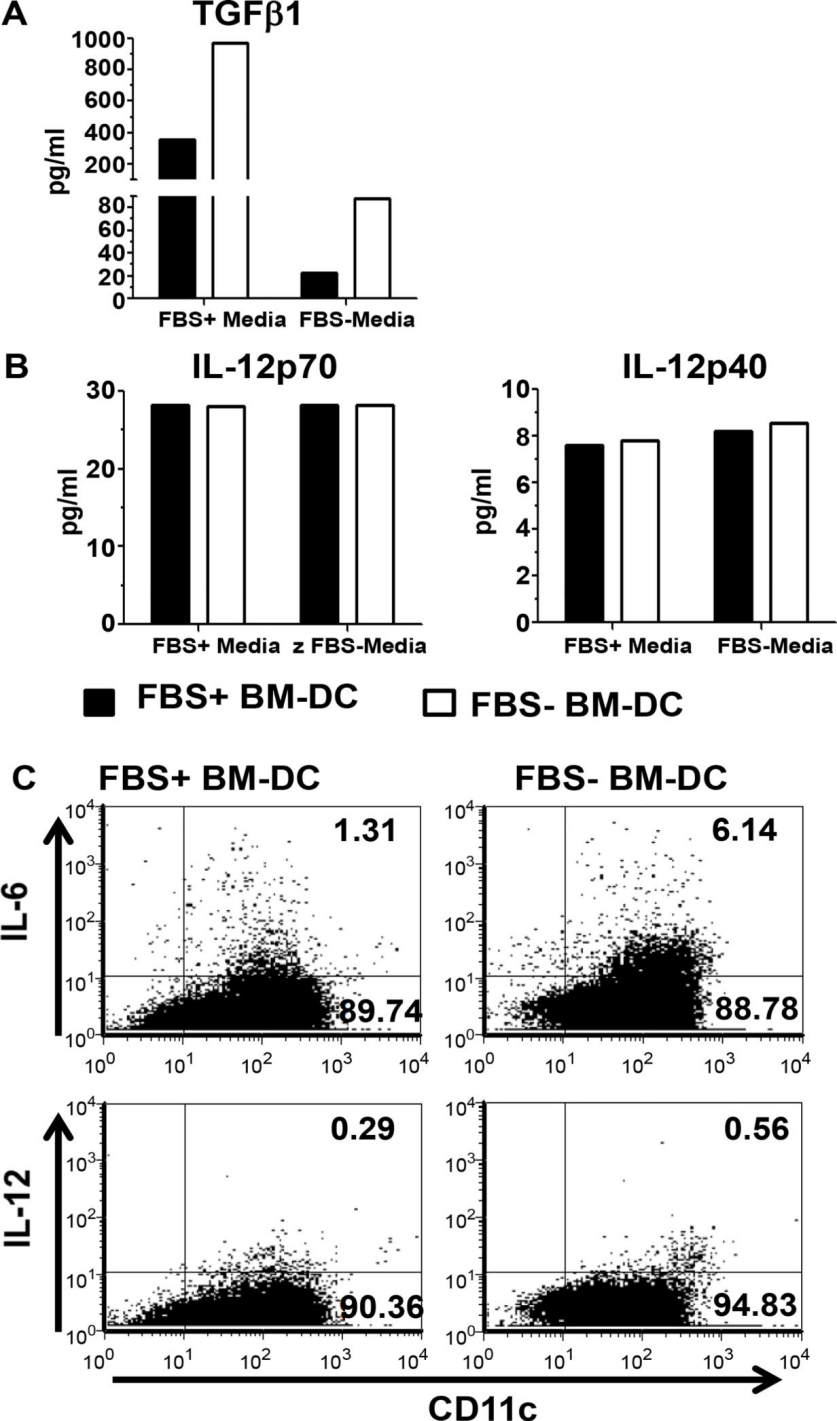
FBS+ or FBS− BM-DC cytokine production. On day 5, 5×10^5^ isolated CD11c^+^ BM-DC was incubated in FBS+ media or FBS− media and supernatants were collected after 24 hours. Cytokines were measured with a multiplex cytokine immunoassay. **A.** TGFβ1 **B.** IL-12p70 and IL-12p40. **C.** On day 7, FBS+ and FBS− BM-DC cultures were assessed for production of IL-12 and IL-6 by intracellular flow cytometry.

Since the tolerogenicity of BM-DC relies on both the expression of co-stimulatory molecules as well as cytokine production, we investigated the expression of TGFβ1 and the IL-12 subunits p70 and p40. On day 5 of culture, CD11c+ BM-DC were isolated from FBS+ and FBS− cultures and incubated overnight in the absence of exogenous stimuli. In order to further assess the influence of FBS on BM-DC cytokine production, each population of BM-DC (FBS+ and FBS− BM-DC) was also incubated overnight in the opposite culture media. Independent of the media used during the overnight incubation period, FBS− BM-DC produce an 2–4 fold increase in TGFβ1 compared to FBS+ BM-DC, but IL-12p70 and IL-12p40 remained unchanged (Figure 2A and B). We were unable to detect IL-6 or IL-10 production in the supernatant on non-TLR stimulated BM-DC (data not shown). Additionally, we observed a 10–15 fold difference in the expression of TGFβ1 dependent on the media the BM-DC were incubated in overnight (Figure 2A). These media dependent differences were not observed with the expression of IL-12 subunits (Figure 2B). Furthermore, we examined IL-12 and IL-6 expression in BM-DC cultures by intracellular cytokine staining by flow cytometry. In accordance with the IL-12 production observed in the supernatant, on day 7 IL-12 expression was similar in both FBS+ and FBS− BM-DC cultures (Figure 2C). However, we observed a greater than 4 fold increase in the percentage of CD11c+IL-6+ cells in the FBS− BM-DC culture (Figure 2C). These results demonstrate that BM-DC expanded in the presence of FBS acquire a distinct phenotype and cytokine production profile as indicated by the increased expression of CD86 and decreased production of TGFβ1 and IL-6 relative to FBS− BM-DC.

### BM-DC induced T cell proliferation and cytokine production

**Figure 3:**
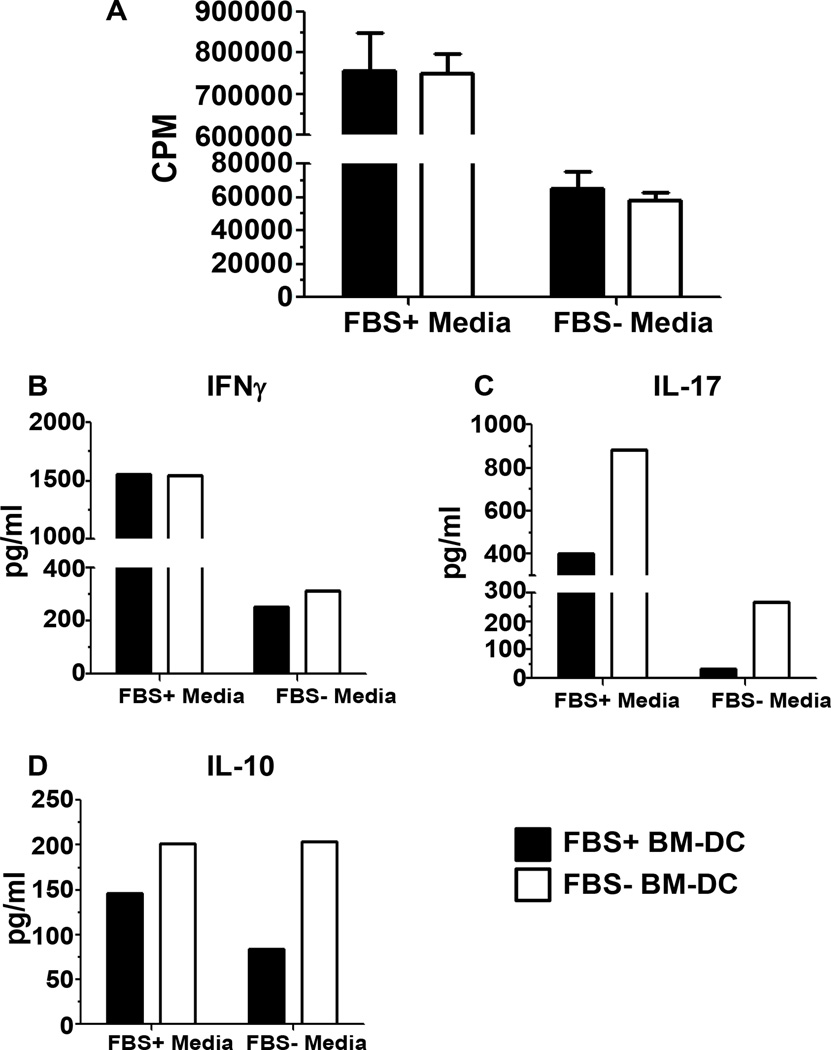
*In vitro* antigen specific T cell proliferation and cytokine production. 1×10^4^ FBS+ or FBS− BM-DC pulsed with 1040-55 mimepitope peptide were used to stimulate 1×10^5^ BDC2.5 CD4^+^ splenocytes in both FBS+ and FBS− culture media for 96 hours. **A.** Tritiated thymidine uptake was measured during the final 18 hours of incubation. **B–D.** Supernatant was removed after 72 hours of incubation and the indicated cytokines were measured with a multiplex cytokine immunoassay.

Since the cytokine profile and phenotype of BM-DC affects the T cell response, we next assessed the ability of FBS+ and FBS− BM-DC to stimulate T cell proliferation and cytokine production *in vitro*. BM-DC from each culture system was incubated with enriched CD4+ T cells from the spleen of TCR transgenic NOD.BDC2.5 mice in the presence of the BDC2.5 mimepitope 1040-55. Due to the significant difference in TGFβ1 production observed between BM-DC cultured in the presence or absence of FBS (Figure 2A), we co-cultured both BM-DC populations (FBS+ and FBS− BM-DC) with CD4+ T cells in both FBS+ and FBS− media to further determine the effect of FBS on DC-T cell interactions. We observed a 10 fold increase in T cell proliferation when the DC-T cell co-culture contained FBS indicating the requirement for FBS for maximal T cell proliferative activity in this type of assay (Figure 3A). Most importantly, there was no difference in the level of T cell proliferation induced by either FBS+ or FBS− BM-DC (Figure 3A) independent of the media used. Therefore BM-DC resulting from culture in FBS+ or FBS− media is equally able to induce the proliferation of T cells in an antigen-specific manner. However, as proliferation is not the sole endpoint for evaluating T cell responses, we further analyzed the DC-T cell co-culture supernatant for the presence of T_h_ cell-related cytokines on Day 3. Concomitant with the observed proliferation, independent of the type of BM-DC (FBS+ BM-DC or FBS− BM-DC), we detected decreased expression of all cytokines in the FBS− co-culture system and this effect was most notable for IFNγ and IL-17 production (Figure 3B–D). Additionally, when comparing cytokine production induced by different BM-DC in the same co-culture system, IFNγ production was similar (Figure 3B). In contrast, FBS− BM-DC induced a 2–9 fold increase in IL-17 production in the FBS+ and FBS− co-culture system, respectively (Figure 3C). IL-10 production also tended to be increased in co-cultures containing FBS− BM-DC though the trend was less notable than IL-17 (Figure 3D). As we were unable to detect IL-10 production when assaying cytokines from non-TLR stimulated BM-DC, it is unlikely that the IL-10 detected in the DC-T cell co-culture system was produced by DC. Additionally, when we assessed IL-10 production from LPS stimulated BM-DC the level of IL-10 production observed was 10 fold less than what was observed in the DC-T cell co-culture (data not shown). We were unable to detect IL-4 in either of the co-culture systems (data not shown) perhaps due to an increased tendency of BDC2.5 CD4 T cells to produce large amounts of IFNγ or differentiate into T_h_1 cells *in vivo* [15–17]. The relative increase in both IL-17 and IL-10 production induced by FBS− BM-DC suggests a change in the T_h_ cell subset induced by BM-DC/T cell interaction.

**Figure 4:**
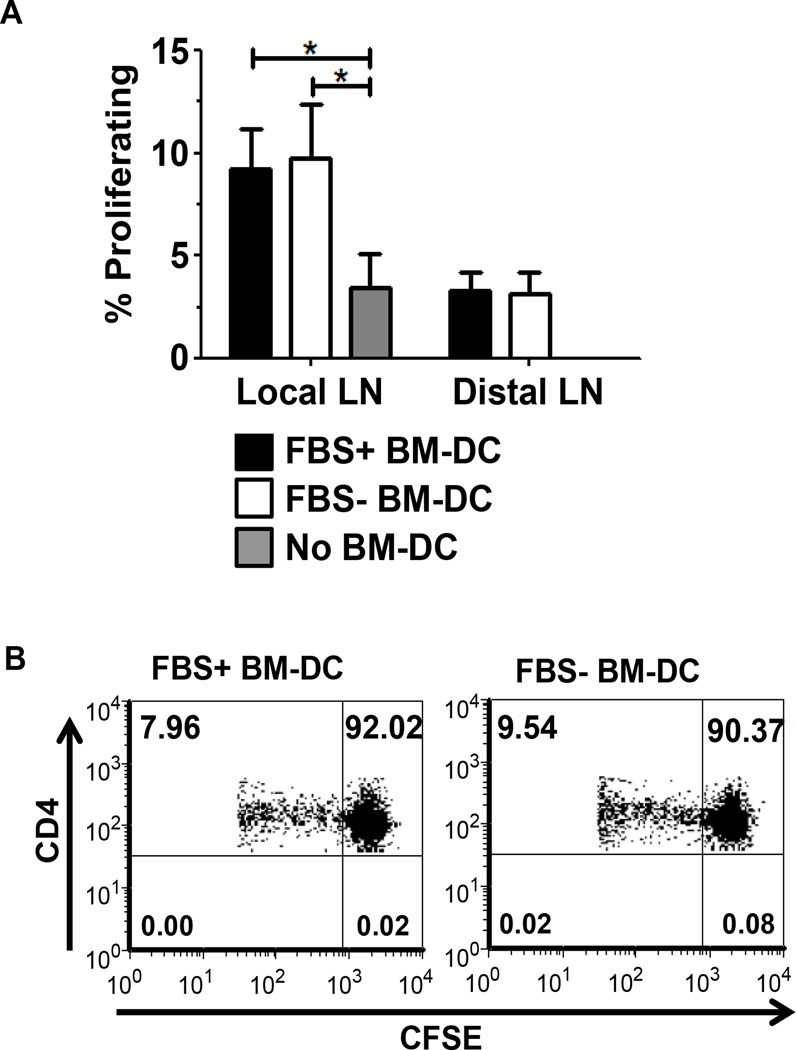
*In vivo* BM-DC-induced CD4^+^ T cell proliferation. Recipient wt NOD females were subcutaneously treated with FBS+ or FBS− BM-DC pulsed with the GAD217-238 peptide or no BM-DC. 20×10^6^ CFSE labeled splenocytes from wt NOD were simultaneously injected intravenously. On day 5 post injection, the popliteal lymph node (Local) and axial lymph node (Distal) were harvested and proliferation of CD4^+^ CFSE^+^ T cells was assessed. A. Results are shown from FBS+ BM-DC (n=5), FBS− BM-DC (n=5) and No BM-DC (n=4). B. Representative dot plots from the Local lymph nodes of FBS+ and FBS− BM-DC treated mice.

As our transgenic *in vitro* experiments suggested that FBS+ and FBS− BM-DC were equally capable of promoting T cell proliferation, we next confirmed their ability to stimulate T cell proliferation *in vivo* in a non-transgenic system. FBS+ and FBS− BM-DC pulsed with GAD217-236 peptide were injected subcutaneously into the footpad of wild type NOD mice. Simultaneously, CFSE-labeled spleen cells from a wild type NOD female were adoptively transferred into the BM-DC recipients or control mice. FBS+ and FBS− BM-DC induced similar CD4 T cell proliferation within the local lymph node (Figures 4A and B). This further confirmed our *in vitro* proliferation study.

### Systemic and islet specific effects of low dose BM-DC treatment

**Figure 5:**
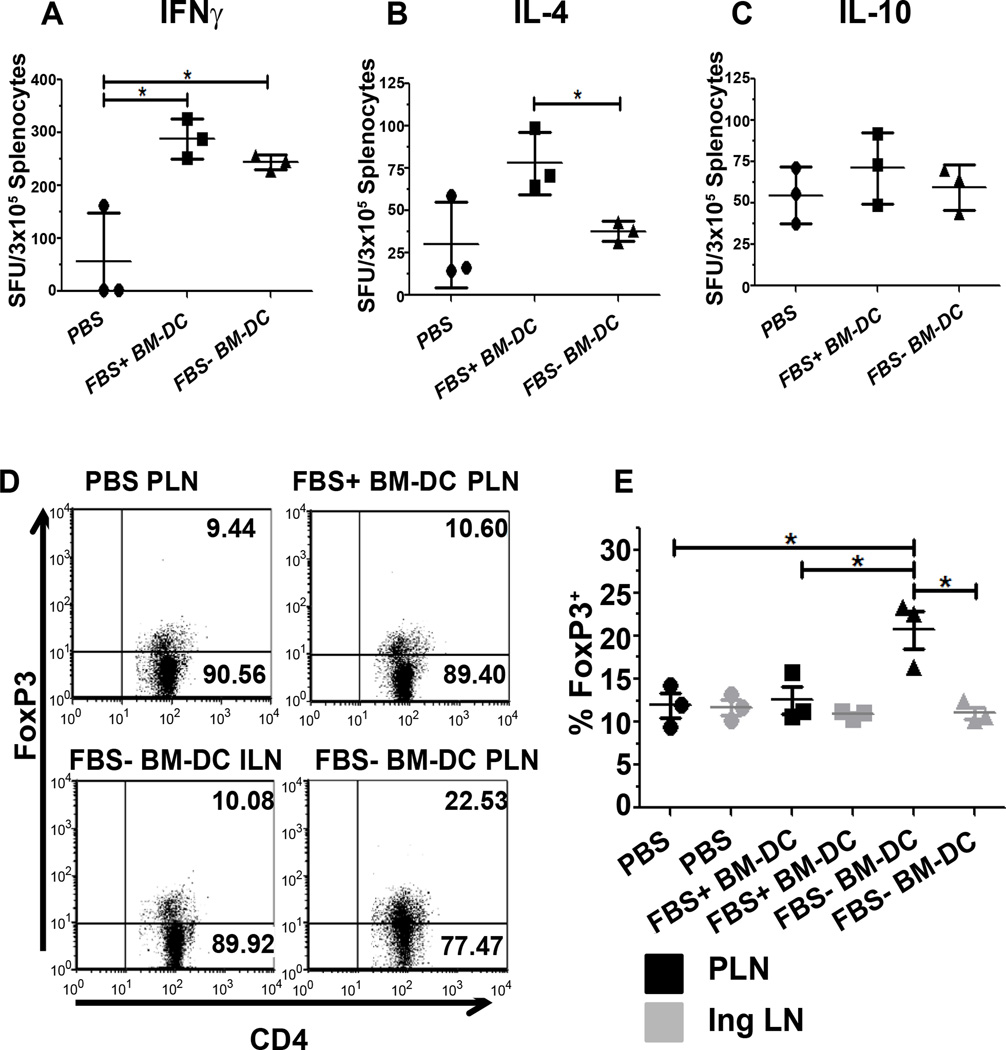
Splenic cytokine producing cells and pancreatic-draining lymph node specific Foxp3 expression. **A–C.** Two weeks following BM-DC treatment, 3×10^5^ spleen cells were re-stimulated with GAD217-236 peptide for 4 days (n=3). Total spot forming units (SFU) were enumerated for the indicated cytokines with an automated spot counter. **D,E.** Two weeks following BM-DC treatment, Inguinal (IngLN) and Pancreatic-draining Lymph Node (PLN) CD4^+^ T cells were examined for Foxp3 expression (n=3). **D.** Representative dot plots of showing Foxp3 production in CD4^+^ T cells.

Due to the differences we observed in cytokine production in the *in vitro* experiments described above, we subsequently investigated the effect of β cell specific FBS+ and FBS− BM-DC treatment on the systemic T_h_ cell response as well as the regulatory T cell response in the pancreatic draining lymph node (PLN). Two weeks following completion of BM-DC treatment, spleen cells from treated or control mice were re-stimulated with GAD217-236 peptide and the number of cytokine producing cells was quantified using an ELI Spot assay. There was no difference in the number of IL-10 producing cells between either of the treatment groups or the PBS-treated control group (Figure 5C). Compared to PBS control mice, IFN γ producing cells were increased following treatment with both FBS+ and FBS− BM-DC (Figure 5A). However, there was no difference in the number of IFNγ producing cells between these two BM-DC treated groups (Figure 5A). In contrast to IFNγ, IL-4 producing spleen cells were increased only with FBS+ BM-DC treatment (Figure 5B). Along with our *in vitro* data, this suggests FBS+ BM-DC initiate a more robust T_h_2-type response whereas FBS− BM-DC induce either a T_h_1 or T_h_17 type response.

In addition to systemic effector T cell populations, we also examined the expression of FoxP3 as a surrogate marker for regulatory T cells within secondary lymphoid organs. Only FBS− BM-DC treatment induced an increase in FoxP3 expression in CD4^+^ T cells within the PLN (Figure 5D and E). This effect was not observed in other control lymph nodes. These experiments further support the notion that including FBS in the culture media in which BM-DC are generated imprints distinct functional characteristics on the resulting DC population that may affect the outcome of T1D DC therapy.

### Prevention of T1D by BM-DC treatment and long term systemic T cell response

**Figure 6:**
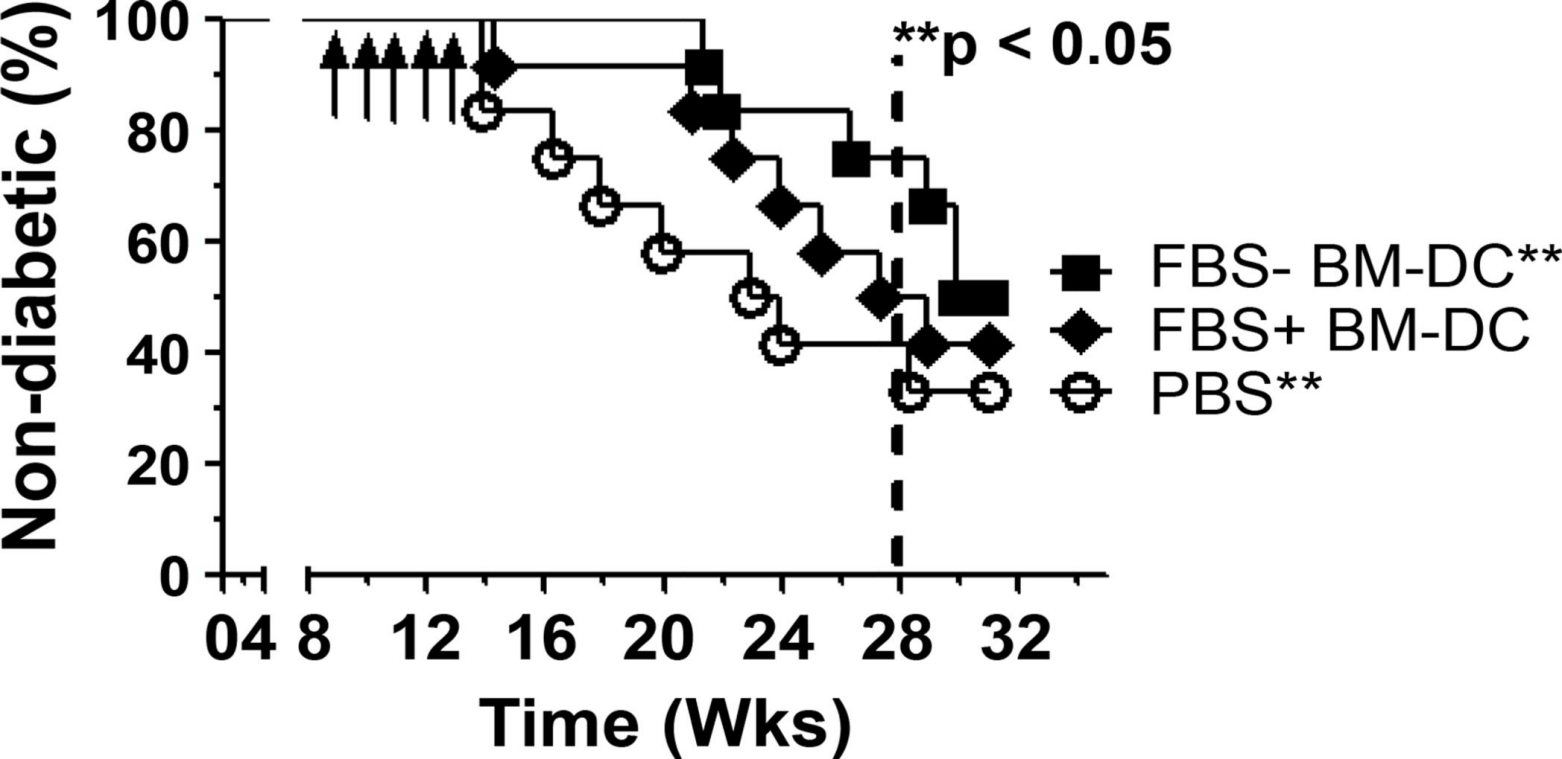
FBS− BM-DC treatment prevents T1D onset. NOD were treated with 1×10^5^ FBS+ BM-DC, FBS− BM-DC or PBS once per week for 5 weeks and T1D onset was monitored for 20 weeks (n=12). Survival curve was analyzed using a Gehan-Breslow-Wilcoxon test.

To determine the effect of FBS+ and FBS− BM-DC on T1D protection, female NOD mice were treated intravenously with GAD217-236 pulsed BM-DC or PBS and monitored for T1D development as described above. Though FBS+ BM-DC delayed T1D onset, only FBS− BM-DC prevented T1D development for 15 weeks following completion of the treatment protocol (Figure 6).

**Figure 7:**
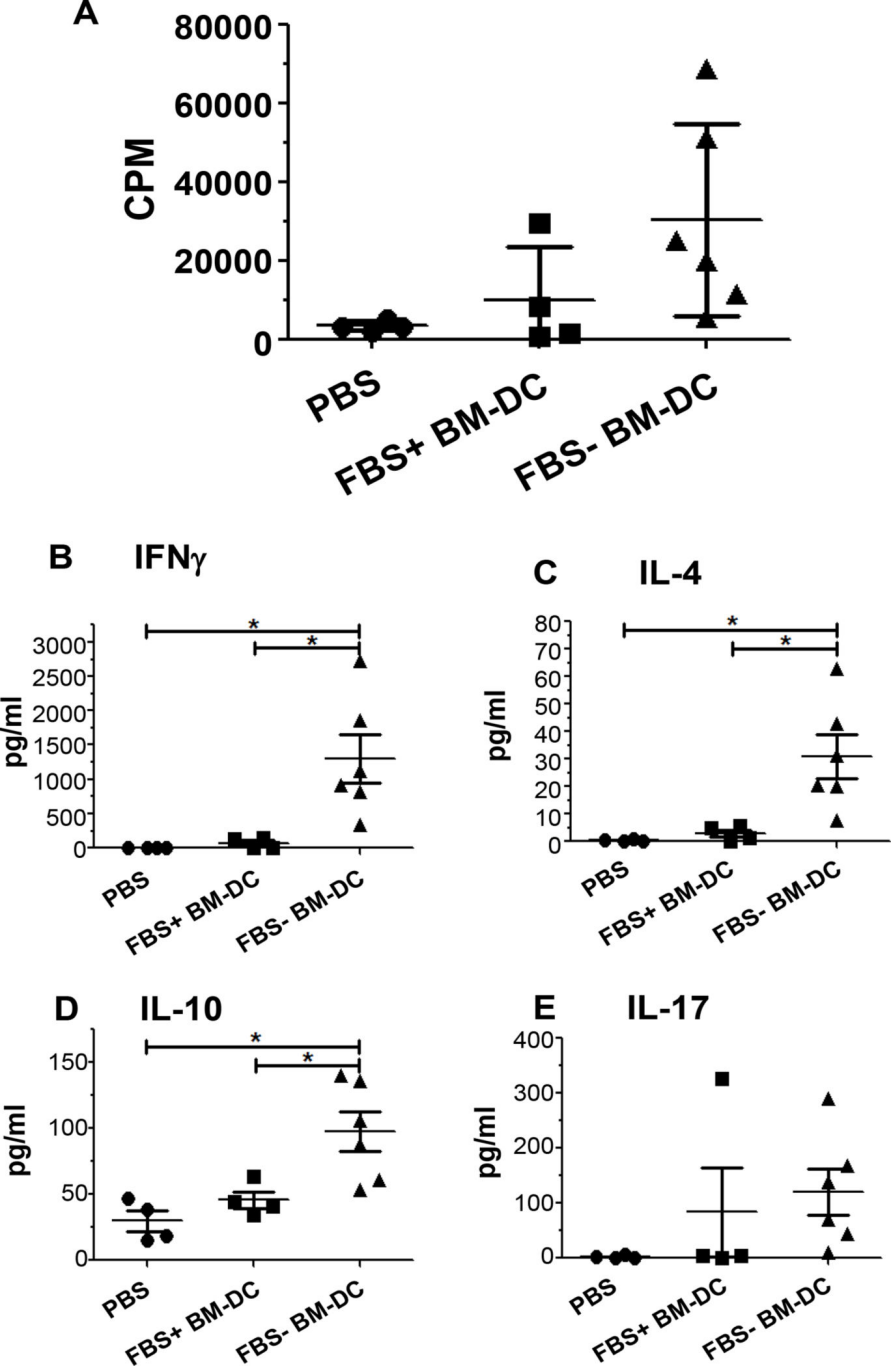
Splenic recall response of non-diabetic mice 20 weeks post BMDC treatment. A–E. 1×10^6^ spleen cells from FBS+ BM-DC, FBS− BM-DC or PBS treated mice were re-stimulated with GAD217-236 peptide for 4 days. **A.** Tritiated thymidine uptake was measured for the final 16 hours of culture. **B–E.** Supernatant was removed after 72 hours of culture and the indicated cytokines were measured with a multiplex cytokine immunoassay.

As the mice in our prevention study reached 30 weeks in age, our cohort of PBS treated mice failed to reach the expected 80% or above incidence of T1D (Figure 6). In light of this fact, we investigated the systemic T cell response to further assess the long term effect of BM-DC treatment. By 20 weeks following completion of treatment (32 weeks in age) a total of 14 mice remained non-diabetic; 4 PBS, 4 FBS+ BM-DC and 6 FBS− BM-DC. Spleen cells from these mice were re-stimulated with GAD217-236 peptide and their subsequent proliferation and cytokine production was assessed. There was no statistical difference in the proliferative capacity of spleen cells from either of the BM-DC treatment groups relative to PBS controls. However, there was a trend towards increased proliferation in both BM-DC treated groups and this trend approached significance in the FBS− BM-DC treated group relative to PBS treatment (p=0.0637; Figure 7A). Nonetheless, the cytokine profile of the re-stimulated spleen cells displayed significant changes between treatment groups. Twenty weeks after the completion of the BM-DC treatment regimen, splenocytes from FBS− BM-DC treated mice produced increased levels of IFNγ, IL-4 and IL-10 relative to splenocyte cultures from PBS and FBS+ BM-DC treated mice (Figure 7B–D). Additionally, an increase in IL-17 production from the splenocytes of FBS− BM-DC treated mice approached statistical significance compared to PBS treated mice whereas FBS+ BM-DC treated mice did not (p=0.0524; Figure 7E). This data highlights the ability of FBS− BM-DC treatment to generate an antigen-specific T_h_ cell population capable of responding to β cell related antigens long after the treatment protocol has ended.

## Discussion

DC represents the command and control center of the adaptive immune system and is thus uniquely situated to confront the T cell mediated destruction of β cells associated with T1D. A pioneering breakthrough in the use of DC came with the advent of *ex vivo* DC expansion; first from the bone marrow of rodent models and subsequently from the blood and bone marrow of human subjects [18–20]. *Ex vivo* expansion of DC is a critical step in the therapeutic employment of DC due to their relative paucity in the peripheral blood [21]. The reliance on FBS in ex vivo DC culture systems, and the inability to directly translate this to human studies, represents a challenge to moving DC therapy from the bench to the bedside. To address this concern, investigators have employed two distinct methods; utilization of autologous serum or use of chemically defined serum free medium. However, there is a large degree of phenotypic and functional variance in the resulting BM-DC populations utilizing both methods [4,10–14]. Specifically, the use of autologous mouse serum has been shown to both increase [13] and decrease [4,14] expression of CD86 relative to BM-DC from FBS containing cultures. Furthermore, the ability of this BM-DC to stimulate T cell proliferation is also inconsistent [13,14]. In addition to the observed differences in phenotype and function, the use of autologous serum, though eliminating FBS associated impediments, also raises concerns about the ability of translating this cell based therapy to the clinic. Pro-inflammatory mediators such as TNFα, IL-8 and IL-1α/β have been shown to be elevated at T1D onset; the same time at which a patient’s own serum would likely be collected for use in expanding DC [22,23]. DC exposure to these cytokines may enhance the immunogenicity of the resulting DC populations with deleterious effects on T1D treatment. Additionally, the inherent differences between patient’s sera may decrease the reproducibility of DC therapy. In light of these concerns, our focus was on the utilization of chemically defined serum free media to culture BM-DC. Though this method may abrogate the concerns of FBS and autologous serum, reports of serum free BM-DC culture have not provided corroborating results [10–12]. Thus, there is a need to further define the conditions necessary to generate BM-DC that provide the highest degree of T1D protection while reducing any potentially negative effects of the culture system. We have provided evidence that BM-DC generated in chemically defined serum free media are phenotypically and functionally distinct, and provide a more therapeutically effective and clinically relevant T1D treatment option relative to BM-DC generated in the presence of FBS.

Our results indicate that NOD BM-DC generated in serum free media were phenotypically distinct from BM-DC generated in the presence of FBS. Additionally, our *in vitro* phenotypic data support the *in vivo* evidence of an immunoregulatory FBS− BM-DC population. Though both culture conditions induced a similar percentage of CD11c^+^CD11b^+^ BM-DC, the expression of CD11b, a heterodimeric integrin involved in cell-cell and cell-ECM contact, was reduced on a per cell basis in FBS− BM-DC. Evidence has suggested that clustering of CD11b induces NF-κB activation, which is generally associated with the activation of monocytes and their subsequent increase in co-stimulatory molecule expression [24]. The large increase in MFI suggests an increased ability to cluster and thus may lead to increased activation of NF-κB and downstream inflammatory pathways. Furthermore, the expression of co-stimulatory molecules, generally considered of more importance to the immunogenicity of DC than CD11b clustering, also varied between BM-DC cultured in the presence or absence of FBS. Specifically, we observed decreased expression of CD86 in FBS− BM-DC but no observable changes in the expression of CD80. CD86 and CD80 are known to be potent co-stimulators in the differentiation of naive T cells into effector cells. Thus a decrease in these molecules implies a more tolerogenic DC phenotype. Additionally, evidence has shown differential effects of the interactions between each of these molecules and their T cell ligands CD28 and CTLA-4. Specifically, CD80/CTLA-4 signaling promotes the suppressive function of regulatory T cells and blocking this interaction accelerates T1D onset [25–28]. The decreased CD86:CD80 ratio observed in serum free BM-DC may indicate a relative preference for induction of suppressive function in regulatory T cells. In support of this, we also observed an increased production of TGFβ1 by FBS− BM-DC relative to FBS+ BM-DC. DC production of TGFβ has been linked to the induction and expansion of regulatory T cells [29–31]. Accordingly, we observed increased FoxP3 expression within CD4^+^ T cells in the pancreatic draining lymph node of FBS− BM-DC treated mice but not in FBS+ BM-DC treated mice. This result suggests that β cell peptide pulsed FBS− BM-DC induced more β cell antigen specific T_regs_ which tended to accumulate in the pancreatic draining lymph nodes. Taken together these data support increased tolerogenicity of BM-DC generated in a serum free culture system.

In addition to the above mentioned characteristics, we also investigated the expression of MHC class II but found no discernable difference between the two BM-DC populations. In light of this, and the observed change in the CD86 to CD80 ratio as well as TGFβ1 and IL-6 expression, we further explored the ability of this BM-DC to stimulate antigen specific T cells. We failed to observe any change in the level of proliferation within the co-culture; however, we did note a change in cytokine production. IL-17 production was increased in the FBS− BM-DC co-culture system suggesting a possible skewing towards a T_h_17 T cell profile. This is supported by the increase in TGFβ1 and IL-6 expression by FBS− BM-DC, both of which are required for the induction of T_h_17 cells. This shift in cytokine production towards a T_h_17-like pattern indicates the ability of FBS− BM-DC to induce T cell responses distinct from FBS+ BM-DC stimulated T cells. Furthermore, recent evidence suggests that a skewing towards a Th17 response and away from a Th1 response may protect from T1D onset [32,33]. Additionally, though this *in vitro* response may seem contradictory to the *in vivo* observation of increased FoxP3-expressing CD4 T cells, reports have shown a high degree of plasticity between regulatory T cells and T_h_17 cells and thus this difference may reflect more on the use of the BDC2.5 TCR transgenic system or the non-physiological *in vitro* conditions [34,35].

The results of our splenic recall experiment two weeks after completion of T1D treatment indicates that both FBS+ and FBS− BM-DC therapy induce a large number of IFNγ producing cells. However, in FBS+ BM-DC treated mice we also observed an increased number of IL-4 producing cells indicative of a T_h_2-type T cell response. This is in agreement with reports by von Herrath et al. of a systemic FBS-specific Th2 response following FBS+ BM-DC treatment but not FBS− BM-DC treatment [14]. In accordance with that study, the results of our prevention study show that FBS+ BM-DC treatment slightly delays T1D onset, though only FBS− BM-DC significantly prevent T1D development. Along with the observed increase in IL-4 producing cells following BM-DC treatment, this supports the notion of a skewing towards an FBS-specific systemic T_h_2 response that slows T1D development.

Lastly, though our data show an above average number of control mice remained non-diabetic beyond 30 weeks of age, it is important to note only the FBS− BM-DC treated mice showed evidence of a persistent β cell specific recall response. The FBS+ BM-DC treatment group displayed virtually no antigen-specific recall response suggesting that the absence of overt T1D pathogenesis in these mice was likely not the result of the treatment regimen. Thus we conclude that FBS− BM-DC are phenotypically more tolerogenic and induce a T cell response that is more protective against T1D onset than FBS+ BM-DC. Our study provides valuable evidence for translating DC prepared in serum free media into clinical applications.

## Abbreviations

BM-DC: Bone Marrow derived Dendritic Cells
FBS: Fetal Bovine Serum
